# DNA Methylation As an Epigenetic Mechanism in the Development of Multiple Sclerosis

**DOI:** 10.32607/actanaturae.11043

**Published:** 2021

**Authors:** I. S. Kiselev, O. G. Kulakova, A. N. Boyko, O. O. Favorova

**Affiliations:** Pirogov Russian National Research Medical University, Moscow, 117997 Russia

**Keywords:** DNA methylation, epigenetics, multiple sclerosis

## Abstract

The epigenetic mechanisms of gene expression regulation are a group of the key cellular and molecular pathways that lead to inherited alterations in genes’ activity without changing their coding sequence. DNA methylation at the C5 position of cytosine in CpG dinucleotides is amongst the central epigenetic mechanisms. Currently, the number of studies that are devoted to the identification of methylation patterns specific to multiple sclerosis (MS), a severe chronic autoimmune disease of the central nervous system, is on a rapid rise. However, the issue of the contribution of DNA methylation to the development of the different clinical phenotypes of this highly heterogeneous disease has only begun to attract the attention of researchers. This review summarizes the data on the molecular mechanisms underlying DNA methylation and the MS risk factors that can affect the DNA methylation profile and, thereby, modulate the expression of the genes involved in the disease’s pathogenesis. The focus of our attention is centered on the analysis of the published data on the differential methylation of DNA from various biological samples of MS patients obtained using both the candidate gene approach and high-throughput methods.

## INTRODUCTION

Epigenetic processes include inherited (at least during mitosis) changes in gene expression that do not affect the DNA nucleotide sequence [1]. However, this classical definition is today often extended to include stable, long-term variations in the cellular transcriptional profile that are not necessarily inherited in the number of epigenetic events [2].

**Fig. 1:**
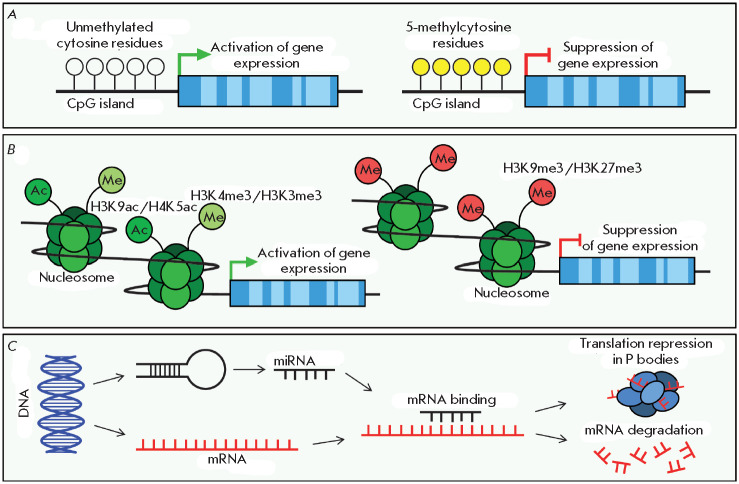
The major epigenetic mechanisms regulating gene expression. The exon-intron structure of a gene is shown as dark blue and light blue rectangles, respectively. (*A*) – Methylation of cytosine residues in the CpG island located in the gene promoter region. (*B*) – The most common modifications of the histone proteins involved in gene expression activation (acetylation of either histone H3 lysine 9 or histone H4 lysine 5 (H3K9ac/H4K5ac) and trimethylation of either histone H3 lysine 3 or histone H3 lysine 4 (H3K4me3/H3K3me3)) and suppression (trimethylation of either histone H3 lysine 9 or histone H3 lysine 27 (H3K9me3/H3K27me3)). (*C*) – MicroRNA-mediated repression of mRNA translation and degradation

The central mechanisms of epigenetic regulation of gene expression are presented in *Fig. 1*. They include DNA methylation (*A*); histone modification, i.e. functionally significant biochemical changes in chromatin that affect the accessibility of certain genomic loci to transcription enzymes (*B*); and the regulation of gene expression at different levels of genetic information implementation with the involvement of regulatory non-coding RNAs, among which the microRNAs regulating expression at the post-transcriptional level are the best studied (*C*) [3].

These mechanisms act synergistically and form a system that regulates the key cellular processes; therefore, they are crucial for a normal development and differentiation of all body cell types [4]. By now, the effect of numerous environmental factors has been proven to be mediated by various epigenetic mechanisms [5]. In some cases, this interaction leads to stable pathological changes that underlie many chronic diseases [6].

Although the investigation of the role of epigenetic mechanisms in the development of common human diseases first focused for the most part on oncological diseases [7], more and more of researchers’ attention is currently focused on different pathologies, in particular autoimmune and neurodegenerative ones [8, 9]. Identification of the features of the epigenetic regulation characteristic of these pathologies can help in our understanding of the mechanisms of their development and contribute to the creation of new effective therapeutic drugs.

In this review, we will focus on one of the key mechanisms of the epigenetic regulation of gene expression, namely DNA methylation, and its role in the development of multiple sclerosis (MS), a socially potent, severe disease of the central nervous system (CNS) characterized by chronic autoimmune inflammation and neurodegeneration.

## MOLECULAR MECHANISMS OF THE EPIGENETIC REGULATION OF GENE EXPRESSION

DNA methylation is a universal epigenetic mechanism that suppresses gene expression in various ways and is involved in the regulation of the activity of the other two mechanisms mentioned above: histone modification and gene expression regulation by non-coding RNAs. In the overwhelming majority of cases, DNA is methylated at the C5 position of cytosine in CpG dinucleotides (CpG sites). The CpG sites that undergo methylation are unevenly distributed throughout the genome; they can form clusters called CpG islands. CpG islands are DNA regions at least 500 bp long with > 55% content of G and C nucleotides and a > 65% ratio of the actual number of CpG sites to the expected one with uniform distribution throughout the genome [10]. CpG islands and neighboring areas (shore) within 2 kb are of the greatest functional significance, since their methylation/demethylation effectively changes the expression level of nearby genes (see *Fig. 1A*). There are also distant areas (shelf) located within 2 kb from the neighboring regions and the rest of the genome (sea), where CpG sites are rare and distributed relatively evenly. About 70% of the gene promoters contain CpG islands [11], which determines the participation of the latter in gene expression regulation.

**Fig. 2:**
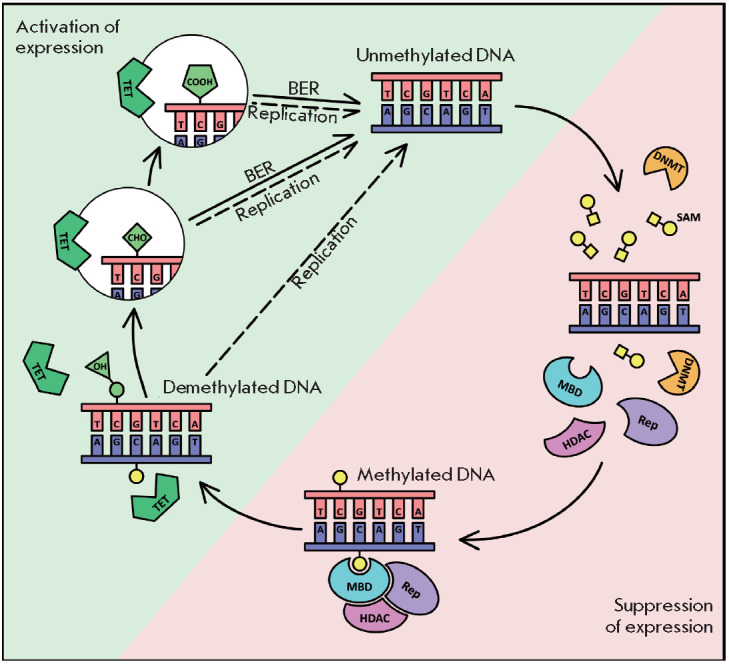
DNA methylation as an epigenetic mechanism of gene expression regulation (see the text for details). BER – base excision repair; DNMT – DNA methyltransferase; HDAC – histone deacetylase; MBD – methyl- binding domain protein; SAM – S-adenosyl methionine; TET – TET methylcytosine dioxygenase; Rep – repressor protein

An overall scheme summarizing our current understanding of the molecular mechanisms of methylation and demethylation of CpG sites in the genome and their involvement in gene expression regulation is shown in *Fig. 2*.

DNA methylation is performed by DNA methyltransferases (DNMTs), enzymes that can transfer a methyl group to the fifth carbon atom of the cytosine residue to form 5-methylcytosine (5mC), using S-adenosyl methionine (SAM) as a donor [12]. The DNMT family includes DNMT1, DNMT2, and the DNMT3 subfamily consisting of DNMT3a, DNMT3b, and DNMT3L. DNMT1 is responsible for DNA methylation after replication and able to rapidly methylate the newly synthesized DNA strand complementary to the template strand. The DNMT3 subfamily is involved in *de novo *DNA methylation [13]. DNMT2/TRDMT1, tRNA (cytosine-5-)-methyltransferase, is technically not a DNA methyltransferase; it is involved in cytosine methylation at the 38 residue of the tRNA anticodon loop.

Methylation of CpG sites in the gene promoter region utilizes methyl-binding domain (MBD) proteins that are capable of suppressing gene expression through two different mechanisms. The first response to promoter methylation is the assembly of MBD-based protein complexes, including corepressor proteins (Rep) that provide rapid suppression of expression by preventing the binding of transcription factors [14]. For long-term stable gene suppression, MBD proteins can recruit histone deacetylases (HDACs) and, thus, initiate another mechanism of epigenetic regulation of gene expression: histone modification leading to chromatin condensation in the gene region [15, 16].

Demethylation of 5-methylcytosine involves TET methylcytosine dioxygenases 1, 2, and 3, which belong to the same family. They can catalyze the oxidation of 5-methylcytosine to 5-hydroxymethylcytosine, then to 5-formylcytosine, and finally to 5-carboxylcytosine [17]. The resulting modified bases are not recognized as methylated ones by the cell molecular machinery and can remain relatively stable, being gradually lost during the synthesis of new DNA molecules in replication. This passive demethylation process is called replication- dependent dilution. In addition, 5-formylcytosine and 5-carboxylcytosine can be actively eliminated independently of replication through their cleavage from the sugar-phosphate backbone of DNA with the participation of thymine DNA glycosylase, with subsequent repair of the break by base excision repair [17].

As mentioned above, DNA methylation is closely related to the epigenetic mechanism of histone modification [16]. The most significant histone modifications include acetylation and methylation (see *Fig. 1B*). Histones are acetylated at lysine residues by histone acetyltransferases; the reverse process is carried out by histone deacetylases. High levels of histone acetylation contribute to less dense chromatin regions and, thus, increased DNA accessibility to chromatin-binding proteins and transcription enzymes, while a low acetylation level has the opposite effect. Methylation of histones at either lysine or arginine residues is catalyzed by histone methyltransferases, and the effect of methylation on chromatin density and, therefore, gene expression depends on the location of the amino acid residue and the number of methyl groups it possesses [18].

MicroRNA-mediated regulation of gene expression (see *Fig. 1C*) also largely depends on the level of DNA methylation, since it is performed at the posttranscriptional level, and the cellular microRNA level depends on the methylation status of their genes [19]. MicroRNAs are small (18–25 nt long) single-stranded non-coding RNA molecules that can complementarily bind to the target gene mRNA. Binding occurs mainly in the 3’-untranslated region of the target gene and triggers a cascade of reactions resulting in suppressed synthesis of its protein product. Full complementarity between a microRNA and its target mRNA upon their binding activates the enzymes of the endonuclease complex and a subsequent degradation of the target mRNA, while incomplete complementarity suppresses translation at either the initiation or elongation stage, cleavage of the mRNA poly-A sequence and translocation of the mRNA to P bodies for subsequent storage or degradation [20].

DNA methylation is the most studied process among the three described mechanisms of epigenetic regulation of gene expression. Considerable evidence indicating the key role of this process in the development of numerous autoimmune and neurodegenerative diseases in humans has been accumulated to date [8, 9]. These pathologies include MS. We will further consider a set of data that analyzes the contribution of DNA methylation to the development of this severe CNS disease.

## EPIDEMIOLOGICAL, CLINICAL, AND ETIOLOGICAL FEATURES OF MULTIPLE SCLEROSIS

MS is a chronic autoimmune disease; its pathogenesis includes demyelination of CNS axons and neurodegeneration and is accompanied by progressive neurological dysfunction [3]. A steady increase in neurological deficit leads to irreversible disability in young, working age patients, which points to the high social and economic toll of the disease. MS is present almost all over the world, but its prevalence varies greatly in different populations. In the Russian Federation, the disease incidence is about 80 cases per 100,000 [21]. The disease’s prevalence is on the increase, which is associated not only with growth in life expectancy and increasing success in the diagnosis of MS, but also with a real increase in its incidence [22].

**Fig. 3:**
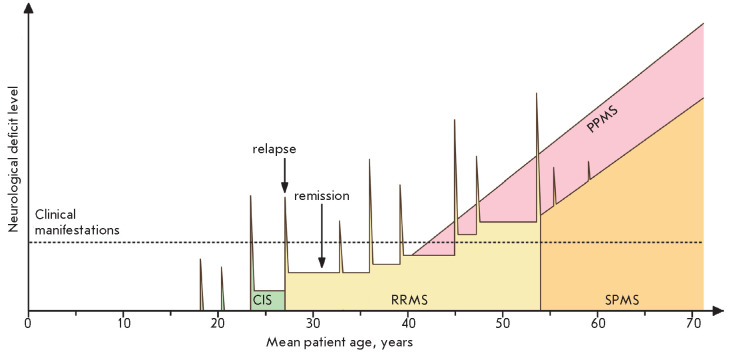
Schematic representation of neurological changes during different clinical courses of MS. The dashed line indicates the neurological deficit level accompanied by clinical manifestations of the disease. The clinically isolated syndrome (CIS) is the first episode of clinical relapse typical of relapsing-remitting MS (RRMS). It can be followed by several years of clinical remission. Repeated relapses followed by periods of remission allow for diagnosing RRMS. Some patients with a prolonged medical history of RRMS develop secondary progressive MS (SPMS) characterized by a steady neurological worsening. Primary progressive MS (PPMS), which is characterized by neurological worsening without remissions from the onset of the disease, is also presented; PPMS manifests itself later than RRMS. The time scale is built based on the mean patient age at the onset of different clinical courses of MS [23, 24, 26]. The number and duration of relapses and remissions are shown schematically

MS is characterized by a pronounced clinical heterogeneity. Most patients have relapsing-remitting MS (RRMS) that is characterized by alternating periods of neurological deficit worsening (relapses) and reduction/ disappearance of neurological symptoms (remissions). In the absence of effective drug therapy, about half of RRMS patients develop secondary progressive MS (SPMS) within 10 years from the onset of the disease, which is characterized by a steady increase in the degree of neurological deficit [23]. A similar clinical picture is observed from the very onset of the disease in 10–15% of patients, and this severe disease course is called primary progressive MS (PPMS) [24]. Different MS courses are characterized by different severities of the autoimmune, inflammatory, and neurodegenerative processes involved in its pathogenesis [25]. The changes in the degree of neurological deficit observed in different MS courses (RRMS, SPMS, and PPMS) are presented in *Fig. 3*.

Like other common autoimmune diseases with a pronounced inflammatory component, MS is generally considered a multifactorial disease; it develops in genetically predisposed individuals upon exposure to environmental factors. The effect of hereditary and external factors can be mediated by epigenetic mechanisms of gene expression regulation, mainly DNA methylation [3].

A fairly large number of environmental factors that can act as disease triggers have been identified so far, and many of them can affect the epigenetic mechanisms of gene expression regulation. These external factors include viral infections. For instance, there is a clear relationship between a high risk of MS and previous infectious mononucleosis, a disease caused by the Epstein–Barr virus [27]. Latent membrane protein 1 (LMP1) of the Epstein-Barr virus is homologous to the surface protein of CD40 B cells and is involved in the stimulation of B-cell-mediated immune and inflammatory responses, thereby increasing the risk of MS and other autoimmune diseases [28]. In addition to the direct stimulation of CD40-dependent signaling pathways, LMP1 can activate the epigenetic mechanism of DNA methylation in cells, which increases the overall methylation level of the host cell genome, resulting in a modulation of the expression of various genes [29].

Another important risk factor for MS is tobacco smoking [30]. Recent studies have shown that, like infection with the Epstein–Barr virus, smoking also stimulates DNA methylation in MS patients [31, 32]. In addition, smoking promotes histone modification and changes in the miRNA expression profiles in a number of cell lines: i.e., it affects all three key mechanisms of the epigenetic regulation of gene expression [33, 34]. The effect of other risk factors for MS, such as the levels of vitamin D [35] and female reproductive hormones [36, 37], on gene expression can also be mediated significantly by epigenetic processes [38, 39, 40].

Significant attention has been historically focused on the investigation of the genetic characteristics of MS. The first genomic region that was shown to be associated with the disease was the HLA major histocompatibility complex genes’ locus. To date, the *1501 allele of the highly polymorphic HLA class II *DRB1* gene is considered the main MS risk marker. Besides this allele, other *DRB1 *variants are associated with the disease in European populations: *0301, *0405, *0801, *1303, etc., as well as a number of alleles of HLA class I genes (HLA-A*0301, HLA-B*3701, *3801, as well as *4402, HLA-C*05, and *07) [41]. Genome-wide association studies (GWAS) proved most efficient in detecting new MS susceptibility markers outside the HLA locus. They have revealed more than 200 disease-associated polymorphic variants to date. At the same time, according to various estimates, the overall contribution of all those identified genetic variants can explain ≤48% of heritability [42]. The epigenetic mechanisms affecting gene expression in various cells and tissues and unrelated to changes in the DNA nucleotide sequence may be key in solving the problem of missing MS heritability.

## STUDY OF DNA METHYLATION IN MULTIPLE SCLEROSIS

Studies of DNA methylation in MS started more than 10 years ago with the use of various approaches, the most common of which were the analysis of the differential methylation of individual candidate genes and genome-wide methylation analysis using high-density DNA microarrays or next generation sequencing (NGS). The DNA methylation analysis of promising candidate genes became the first approach to be used, since it was the most accessible. In the majority of those studies, the analysis was performed using either pyrosequencing or MALDI-TOF mass spectrometry of DNA amplification products after DNA bisulfite conversion, as well as methylation-specific PCR, followed by a comparison of average CpG methylation levels in the studied fragments. Generally, RRMS patients were studied and the control groups consisted of healthy individuals. These studies were few (only 16 articles have been found) and were carried out using DNA obtained from whole blood, its fractions, and brain tissue (*Table 1*).

**Table 1:** Data on DNA methylation in MS patients obtained using the candidate gene approach

DNA source	Study group	Main result	Year [ref.]
T lymphocytes	RRMS patients, control group	Hypermethylation in the alternative VDR promoter in RRMS patients	2017 [43]
	RRMS patients, control group	Hypermethylation in the IL2RA promoter in RRMS patients	2017 [44]
PBMCs	RRMS patients, control group	An association between hypermethylation of LINE-1 retrotransposons and a high risk of RRMS with a low effectiveness of IFN-beta therapy was found	2017 [45]
	RRMS patients, control group	IL2RA gene analysis revealed no differences in its methylation status between study groups	2017 [44]
	RRMS patients, control group	Hypermethylation of TET2 and DNMT1 gene promoters was detected in RRMS patients; there were no significant differences in global methylation	2014 [46]
	RRMS patients, control group	Hypermethylation of the PTPN6 promoter in RRMS patients	2012 [47]
	RRMS patients, control group	Analysis of PADI2 and PADI4 showed hypomethylation of the PADI2 promoter in RRMS patients	2012 [48]
	Monozygotic twins discordant for MS	Analysis of CIITA revealed no differences in the methylation status between groups	2008 [49]
Whole blood	RRMS patients	Hypomethylation of BDNF in patients with higher disease progression rates	2018 [50]
	RRMS patients during relapse and remission, control group	Analysis of RUNX3, MLH1, IGF2, CDKN2A, SOCS1, NEUROG1, CACNA1G, and CRABP1 showed differential methylation of RUNX3, CDKN2A, SOCS1, and NEUROG1 in RRMS patients compared to controls; there were no differences between relapse and remission patients	2018 [51]
	RRMS patients, control group	Analysis of TMEM39A revealed no differences in the methylation status between the study groups	2017 [52]
	RRMS patients, control group	Hypermethylation of LINE-1 retrotransposons was detected in patients; methylation level correlated with the average disability score according to the EDSS	2016 [53]
	RRMS and PPMS patients	Analysis of HLA-DRB1*1501 and HLA-DRB5 found no association between their methylation status and clinical MS course	2010 [54]
Blood serum	RRMS patients, control group	Hypermethylation of some L1PA2 members of LINE-1 retrotransposons in RRMS patients	2018 [55]
	RRMS patients during relapse and remission, control group	Hypermethylation of MOG in RRMS patients during relapse compared to remission patients and the control group	2016 [56]
	RRMS patients during relapse and remission, control group	Analysis of a panel of 56 genes revealed significant differences in their methylation levels between all three groups	2010 [57]
Brain tissues	RRMS patients, control group	Analysis of IL2RA showed no relationship between its methylation status in different study groups	2017 [44]
	RRMS patients, control group	Hypermethylation of PADI2 in normal white matter of RRMS patients	2007 [58]

As can be seen from *Table 1*, differential methylation of the genes involved in the regulation of autoimmune responses (*IL2RA*, *PTPN6*, and *SOCS1*) [44, 47, 51] and CNS function (*PADI2*, *CDKN2A*, *RUNX3*, *NEUROG1,* and *BDNF*) [48, 50, 51] was detected in the whole blood and various leukocyte populations of RRMS patients. The observed differences in DNA methylation levels turn out to be divergent, indicating the involvement of this epigenetic process in both the activation [47, 51] and suppression of inflammatory responses in the CNS [44, 48, 50]. Hypermethylation of the *VDR *gene, which codes for the vitamin D receptor whose deficiency is considered one of the key non-hereditary triggers of MS, as well as the *DNMT1 *and *TET2 *genes involved in DNA methylation and demethylation, respectively, was noted in the blood cells [43, 46].

A study of a set of 56 genes in serum-circulating DNA revealed differences in these genes’ methylation levels, which allow for distinguishing RRMS patients during relapses from patients in remission and healthy individuals of the control group with > 70% sensitivity and specificity [57]. Another study showed hypermethylation of *MOG*, which encodes one of the myelin sheath proteins, in the serum of RRMS patients [56]. According to the authors, this may indicate impaired expression of *MOG *in oligodendrocytes, whose DNA enters the bloodstream after their destruction by demyelination. An analysis of brain tissues demonstrated hypomethylation of the peptidyl arginine deiminase type 2 (*PADI2*) gene that is involved in the post-translational modification of the key myelin sheath protein in neurons; namely, the myelin basic protein (MBP) [58]. The fact that this gene is also hypomethylated in the peripheral blood mononuclear cells (PBMCs) of RRMS patients may be an indication of the involvement of the regulatory mechanisms, which are similar among different tissues, in gene expression modulation [48].

The only study comparing the methylation levels of HLA-*DRB1 *and *HLA-DRB5 *in the whole blood of RRMS and PPMS patients [54] found no significant differences between these groups.

Studies of the methylation level of LINE retrotransposons should be mentioned separately. Under normal conditions, these repeated sequences contain many methylated CpG sites, which prevents the transcription of their genes [59]. Therefore, analysis of their differential methylation is a simple way to assess the global level of genome methylation in various tumors and some autoimmune diseases [55]. The methylation level of LINE-1 family retrotransposons was analyzed in RRMS patients in PBMCs, whole blood, and blood serum: LINE-1 hypermethylation was observed in all cases [45, 53, 55]. In addition, an association was found between a greater methylation level of LINE-1 and both severe disability according to the EDSS score and a low efficacy of IFN-beta therapy for RRMS [45, 53]. A good reproducibility of the data on the hypermethylation of LINE-1 elements in MS patients, as well as an association between their methylation levels, disease severity, and drug therapy effectiveness, is an indication that LINE-1 retrotransposons could become promising diagnostic and prognostic markers of MS.

In general, the data obtained using the candidate gene approach have shown that DNA methylation is involved in MS pathogenesis and they paved the way for the investigation of this epigenetic mechanism of gene expression regulation in MS patients using less sensitive, but much more efficient, genome-wide methods. The use of these methods, which primarily include high-density DNA microarrays and NGS, allows for the detection of differentially methylated sites (DMSs), individual CpG sites whose methylation levels change in MS, throughout the genome. *Table 2* summarizes the results of genome-wide studies of DNA methylation in MS patients using different groups for comparison.

It is important to note that the threshold of statistical significance (*p*) for DMS detection at the genome-wide level greatly varies between different studies. In five out of 18 works presented in *Table 2*, corrections for multiple comparisons were applied and the differences were considered significant at *pFDR * < 0.05 [32, 60, 61, 62, 63]. Other studies used a less stringent threshold of statistical significance: a nominal *p *value in a range of 0.05–0.0005. In addition to the *p *value, the minimum difference in the mean CpG methylation level between the compared groups (β), which most often varies within 5–10%, is also used as a selection criterion for DMS [64, 65]. Since DMSs not meeting the criteria selected by the authors are often omitted in publications, we will further rely on the *p *and β values the authors used for DMS detection.

Various blood cells and fractions (whole blood, serum, PBMCs, CD4^+^ and CD8^+^ T cells, CD19+ B cells, and CD14+ monocytes) were mostly used as a source of DNA in the published papers; brain tissue has been studied in only a few works. In some papers, twins discordant for MS were studied. However, in most cases, RRMS patients were compared to unrelated healthy individuals. A few studies analyzed a change in DNA methylation in RRMS patients during therapy using various drugs, during relapse and remission, as well as when comparing RRMS individuals with SPMS and/ or PPMS groups.

**Table 2:** Data on DNA methylation in MS patients obtained using high-throughput methods

DNA source	Study group	Main result	Year [ref.]
CD4+ T lymphocytes	Monozygotic twins discordant for MS (combined group of RRMS, SPMS, and PPMS patients)	Differential methylation of FIRRE	2019 [61]
	RRMS patients, control group	Differential methylation in MOG/ZFP57, HLA-DRB1, NINJ2/LOC100049716, and SLFN12 genes	2019 [66]
	RRMS and SPMS patients, control group	Hypermethylation of the last exons of VMP1/MIR21 in RRMS patients compared to the control group and SPMS patients	2018 [67]
	RRMS patients before and after treatment with dimethyl fumarate	A total of 945 DMSs, 97% of which were hypermethylated after treatment, were found; DMSs of SNORD1A, SHTN1, MZB1, and TNF were located in the promoter region	2019 [64]
	RRMS patients, control group	Differential methylation of the HLA locus in the region of HLADRB1, HLA-DRB5, and RNF39; DMSs were also found in the region of HCG4B, PM20D1, and ERICH1	2017 [65]
	RRMS patients, control group	There were no significant differences in DNA methylation between RRMS patients and healthy controls	2015 [60]
	RRMS patients, control group	Differential methylation of the HLA locus (19 DMSs in the region of HLA-DRB1 and 55 DMSs beyond it); many of them are located within genes whose association with MS had been previously shown	2014 [68]
	Monozygotic twins discordant for MS	There were no significant differences in DNA methylation between twins	2010 [69]
CD8+ T lymphocytes	RRMS patients, control group	Differential methylation of HLA-DRB1 and SLFN12 in RRMS patients; global DNA hypermethylation	2019 [66]
	RRMS patients, control group	A total of 79 DMSs, none of which was located within HLA-DRB1	2015 [70]
	RRMS patients, control group	DNA hypermethylation was found in RRMS patients compared to the control; no differences in methylation levels of individual DMSs were noted	2015 [60]
CD19+ B lymphocytes	RRMS patients during treatment, control group	Multiple DMSs were found within LTA and in the region of PC-associated genes SLC44A2, LTBR, CARD11, and CXCR5	2018 [71]
CD14+ monocytes	RRMS patients, control group	Two DMSs in HLA-DRB1	2018 [72]
CD4+, CD8+, CD19+, and CD14+ leucocytes	RRMS and SPMS patients, control group	DNA methylation levels were assessed separately in CD4+, CD8+, CD19+, and CD14+ cells, followed by selection of DMSs that are universal for different cell types. RRMS- and SPMS-specific methylation patterns were identified	2018 [73]
PBMCs	Monozygotic twins discordant for MS (combined group of RRMS, SPMS, and PPMS patients)	Differential methylation of TMEM232 and ZBTB16 was observed in MS patients and then replicated in an independent sample. IFN-beta therapy induces hypomethylation of RSAD2, MX1, and PLSCR1	2019 [61]
	RRMS and PPMS patients, control group	DNA hypermethylation was found in PPMS patients compared to both RRMS and control groups; 30 and 67 DMSs were detected inRRMS and PPMS compared to the control, respectively; 51 DMSs were found when comparing two MS forms with each other	2016 [74]
Whole blood	RRMS patients, control group	The relationship between smoking and DNA methylation level was found in RRMS patients. The differences were more significant for women and carriers of MS risk haplotypes in the HLA locus	2017 [32]
	RRMS patients, control group	There were no significant differences in DNA methylation between RRMS and control patients	2015 [60]
Brain tissues	RRMS patients, control group	Global DNA hypermethylation and 2,811 individual DMSs were detected in RRMS patients	2019 [62]
	Demyelinated and healthy brain tissue of RRMS patients	Differential methylation of 16 genes, whose expression is characteristic of astrocytes and neurons, was found in the demyelinated hippocampal tissue	2017 [75]
	RRMS patients, control group	Hypermethylation of genes involved in maintaining the vital activity of oligodendrocytes and hypomethylation of genes involved in proteolytic processes were detected in MS patients	2014 [63]

In *Table 2*, the data obtained when analyzing DNA methylation profiles in pairs of monozygous twins discordant for MS should be discussed separately from the other results. Comparison of DNA methylation levels in the CD4^+^ T cells of twins discordant for MS revealed no significant differences in any of the three pairs studied: the number of DMSs observed when comparing twins from each pair was lower than that obtained when comparing unrelated healthy individuals [69]. A study of the same lymphocyte population revealed differential methylation of the *FIRRE *gene between twins, while the analysis of DNA methylation in PBMCs showed the presence of DMSs in the regions of *TMEM232 *and *ZBTB16 *[61]. However, since the study group included patients with RRMS, SPMS, and PPMS, the detected DMSs can be considered only epigenetic markers characteristic of MS in general. It is safe to state that the studies carried out using the twin methods have not led to any unambiguous conclusions so far.

As seen from *Table 2*, most of the published works compared DNA methylation levels in T cells (primarily CD4^+^) between RRMS patients and the control group; however, contradictory data were obtained in most of the cases. In particular, the results of six studies performed using CD4^+^ T cells can be compared to each other. For instance, a study by S.D. Bos *et al*. showed no significant differences in DNA methylation between RRMS individuals and healthy donors [60]. In a study by B. Rhead *et al*., DMSs were found in RRMS patients in the *MOG*/*ZFP57*, *HLA*-*DRB1*, *NINJ2*/* LOC100049716*, and *SLFN12 *genes [66]. S. Ruhrmann* et al*. detected DMSs clusters in the last two exons of the *VMP1*/*MIR21 *gene [67]. Another two studies carried out by the same research group also revealed significant differences in methylation profiles between RRMS patients and healthy individuals [65, 68]. The only differentially methylated region identified in both works was the HLA locus, which turned out to be hypermethylated in patients, mainly in the *HLA*-*DRB1* region, while the markers of differential methylation outside the HLA locus found in [65] and [68] differed between each other and were not identified in other studies.

The results obtained in three works on DNA methylation in CD8^+^ T cells are also difficult to compare. S.D. Bos *et al*. noted global DNA hypermethylation in RRMS individuals; however, no significant differences in the methylation of individual CpG sites were found [60]. The data on global DNA hypermethylation were confirmed by B. Rhead *et al*., who detected DMSs in the region of *HLA*-*DRB1 *and *SLFN12 *in RRMS patients [66]. No trend towards global hypermethylation was observed in a study by V.E. Maltby *et al*.; however, 79 separate DMSs were detected throughout the genome, none of which were located within either *HLA*-*DRB1* or *SLFN12 *[70].

An analysis of CD19^+^ B cells revealed a DMS cluster in the *LTA *gene, and a number of DMSs in the* SLC44A2*, *LTBR*, *CARD11*, and *CXCR5 *genes, which, according to GWAS, are associated with MS [71]. It should be noted that the RRMS group was heterogeneous in that study: it included both patients without drug therapy and patients taking various immunomodulatory drugs. Reduced methylation of *HLA*-*DRB1 *was observed in the CD14^+^ monocytes of RRMS patients, mainly in DRB1*1501 allele carriers [72].

Special attention should be paid to a recent comprehensive study that evaluated the levels of DNA methylation in all mentioned populations of blood leukocytes (CD4^+^ and CD8^+^ T cells, CD19^+^ B cells, and CD14^+^ monocytes) in RRMS and SPMS patients, as well as in healthy individuals in the control group, followed by a selection of DMSs common to different cell types [73]. This significantly increased the power of the statistical analysis and allowed for the identification of methylation patterns specific to RRMS and SPMS patients, which were then validated in DNA samples from CD14^+^ monocytes and the whole blood of independent groups of patients and healthy individuals. Although the use of these integrated approach does not allow for a detailed analysis of the role of DNA methylation in the functioning of individual populations of blood leukocytes during disease development, it helps to identify the features of DNA methylation that characterize different clinical forms of MS. This may be useful for their differential diagnosis at early disease stages; in addition, it also provides clues as to the development of new drugs that are highly effective in the therapy of MS forms poorly responsive to treatment.

PBMCs, a fraction of blood cells mostly consisting of all the previously mentioned subpopulations of leukocytes, can be used as a more accessible object for the search for DNA methylation markers characteristic of different MS forms. We carried out research using the case-control design and analyzed DNA methylation levels in the PBMCs of RRMS and PPMS patients. This analysis showed preferential hypermethylation of PBMC DNA in PPMS patients compared to both RRMS individuals and the control group, and it also revealed a set of individual DMSs specific to each of the studied MS forms [74]. This is the only genome-wide study performed in PPMS patients so far, and its data undoubtedly require validation in independent samples.

Very sparse studies on DNA methylation before and after a course of therapy with immunomodulatory drugs should be mentioned also. DNA isolated from the CD4^+^ T cells of the same RRMS patients was shown to have many DMSs throughout the genome associated with treatment, 97% of which were hypermethylated after treatment [64]. N. Souren *et al*. showed that intake of IFN-beta by patients induces hypomethylation of* RSAD2*, *MX1*, and *PLSCR1 *in PBMCs [61]. Although these data require independent confirmation, they are indicative of an important role for the epigenetic mechanism of DNA methylation in the development and suppression of a MS pathology. In addition, they show the importance of taking into account not only the type of MS course [74], but also the intake of immunomodulatory drugs [64] when selecting homogeneous groups of MS patients for a DNA methylation analysis.

Only a few works [32, 60] used serum and whole blood as a biological source to search for differential methylation profiles characterizing MS, since a wide range of different body cells can be the source of DNA entering the bloodstream, and the observed changes in DNA methylation levels are difficult to interpret. S.D. Bos *et al*. revealed no significant differences in DNA methylation profiles in whole blood samples [60]. Another work showed an association between smoking and the DNA methylation level in the whole blood of RRMS patients, with the most significant differences being found in women and carriers of MS risk haplotypes of the HLA locus [32]. To date, there are only three studies that have analyzed DNA methylation in various brain tissues in MS. Individual DMSs were identified when comparing demyelinated and normal hippocampus tissue in MS patients [75], as well as the white matter [62] and the frontal cortex [63] of RRMS patients and the control group. Differences in the design of studies and in the biological source of DNA do not allow us to reliably compare the results of these works.

In general, despite a rather extensive amount of accumulated data, the HLA gene locus is the only genomic region whose differential methylation in the same biological source has been confirmed in independent studies [65, 66]. Meanwhile, the results obtained in [73] show that universal patterns of differential DNA methylation (at least in different populations of blood leukocytes) can exist in MS. Based on this data, we searched for DMSs identified in more than one study using both the candidate gene approach (*Table 1*) and the high-throughput DNA analysis (*Table 2*), regardless of which leukocyte populations were used as a DNA source. The identified genes and the main functions of their protein products are presented in *Table 3*.

**Table 3:** Genes differentially methylated in MS in different populations of blood leukocytes according to the data of at least two independent studies and the biological functions of their protein products according to the UniProt [76] and NCBI Gene [77] databases

Gene	Biological function of the protein product	Reference
AHRR	Aryl hydrocarbon receptor repressor; it is involved in metabolism of xenobiotics and regulation of cell growth and differentiation	[68, 73]
ATP11A	The catalytic component of the P4-ATPase flippase complex, which ensures the maintenance of asymmetric distribution of phospholipids in membranes	[73, 74]
DYDC2	Protein product can participate in molecular organization of synapses and nerve cell signaling	[70, 73]
DYDC2	Unknown	[70, 73]
ERICH1	Unknown	[65, 73]
GNG7	The gamma subunit of the G protein; it is involved in signaling in adenylate cyclase-dependent pathways in certain brain regions	[68, 73]
HLA-DQB1	Protein product is involved in presentation of antigenic peptides to CD4+ T lymphocytes as part of MHC class II molecules	[68, 73]
HLA-DRB1	Protein product is involved in presentation of antigenic peptides to CD4+ T lymphocytes as part of MHC class II molecules	[65, 66, 68, 72]
HLA-DRB5	Protein product is involved in presentation of antigenic peptides to CD4+ T lymphocytes as part of MHC class II molecules	[65, 68, 73]
HOXC4	Transcription factor involved in cell positioning along the anteroposterior body axis during ontogenesis	[73, 74]
TNXB	Protein product mediates the interaction between the cells and the extracellular matrix	[70, 73]
USP35	Protein product is involved in suppression of NF-kB and inhibition of PARK2-mediated degradation of mitochondria	[68, 73]
ZFYVE28	Negative regulator of epidermal growth factor receptor signaling	[73, 74]

The genes presented in *Table 3* are involved in the immune response (*HLA*-*DQB1*, *HLA*-*DRB1*,* HLA*-*DRB5*, and *USP35*), signal transduction (*AHRR, ATP11A, GNG7, HOXC4, *and *ZFYVE28*), and the interaction with the matrix (*DLGAP2, TNXB*). The role of the *DYDC2 *and *ERICH1 *genes remains unknown. Most of the listed genes were identified in [73] as MS markers universal for different leukocyte populations, which is indicative of their contribution to MS pathogenesis at the level of the integral systems regulating a cell’s vital activity, which are common among different cell types. Differential methylation of *HLA*-*DRB1 *in MS was observed in four studies in CD4^+^ and CD8^+^ T-lyphocytes, as well as CD14+ monocytes [65, 66, 68, 72]. Although the authors of [73] did not consider DMSs in *HLA*-*DRB1 *as MS-associated ones, other HLA genes were included in this category: *HLA*-*A*, *HLA*-*H*,* HLA*-*J*, *HLA*-*DRA*, *HLA*-*DQB1*, and *HLA*-*DRB5*. In addition, *HLA-DRB1 *was found among the markers of differential methylation characteristic of SPMS [73].

HLA genes are believed to play a leading role in genetic predisposition to MS, and the level of significance of the association between the *HLA*-DRB1*15 allele and MS development in GWAS studies exceeds* p * < 5 × 10-1000 [42]. Thus, the fact that, of the more than 200 GWAS-identified MS risk genes differences in methylation levels in at least two independent studies were shown for only HLA genes seems quite indicative. In most cases, during disease development, the effects of DNA methylation and genetic variability apparently manifest themselves through different gene sets, a fact that determines the relative independence of these processes from each other. In addition, DNA methylation almost never affects the master genes but exerts a small effect on the expression levels of many other genes.

In conclusion, the data obtained to date indicate the involvement of the epigenetic mechanism of DNA methylation in MS, which takes place in various blood cells and brain tissues. Further expansion of the list of known genes undergoing epigenetic regulation in MS will make a significant contribution to our understanding of the disease’s pathogenesis. In addition, we may expect the identification of the genes whose methylation levels either differ in different MS courses or change upon exposure to immunomodulatory drugs, which may facilitate the development of effective prognostic tests and the identification of new therapeutic targets.

## Abbreviations

BER: base excision repair
CIS: clinically isolated syndrome
CNS: central nervous system
DMS: differentially methylated CpG-site
DNMT: DNA methyltransferase
EDSS: expanded disability status scale
GWAS: genome-wide association study
HDAC: histone deacetylase
MBD: methyl-binding domain protein
MS: multiple sclerosis
NGS: next generation sequencing
PBMCs: peripheral blood mononuclear cells
PPMS: primary progressive multiple sclerosis
RRMS: relapsing-remitting multiple sclerosis
SAM: S-adenosyl methionine
SPMS: secondary progressive multiple sclerosis
TET: TET methylcytosine dioxygenase
Rep: repressor protein

## References

[1] Dupont C., Armant D.R., Brenner C.A. (2009). Semin. Reprod. Med..

[2] (2020).

[3] Oksenberg J.R. (2013). Expert. Rev. Neurother..

[4] Das A. (2016). Epigenetics, the Environment, and Children’s Health Across Lifespans. Berlin: S.

[5] Alegría-Torres J.A., Baccarelli A., Bollati V. (2011). Epigenomics..

[6] Olden K., Freudenberg N., Dowd J., Shields A.E. (2011). Health Aff..

[7] Kanwal R., Gupta S. (2010). J. Appl. Physiol..

[8] Surace A.E.A., Hedrich C.M. (2019). Front. Immunol..

[9] Urdinguio R.G., Sanchez-Mut J.V., Esteller M. (2009). Lancet Neurol..

[10] Takai D., Jones P.A. (2002). Proc. Natl. Acad. Sci. USA..

[11] Saxonov S., Berg P., Brutlag D.L. (2006). Proc. Natl. Acad. Sci. USA..

[12] Moore L.D., Le T., Fan G. (2013). Neuropsychopharmacology..

[13] Xu F., Mao C., Ding Y., Rui C., Wu L., Shi A., Zhang H., Zhang L., Xu Z. (2010). Curr. Med. Chem..

[14] Choy M.K., Movassagh M., Goh H.G., Bennett M.R., Down T.A., Foo R.S.Y. (2010). BMC Genomics..

[15] Jones P.L., Veenstra G.J., Wade P.A., Vermaak D., Kass S.U., Landsberger N., Strouboulis J., Wolffe A.P., Jan Veenstra G.C., Wade P.A. (1998). Nat. Genet..

[16] MacDonald J.L., Roskams A.J. (2009). Prog. Neurobiol..

[17] Bochtler M., Kolano A., Xu G.L. (2017). Bioessays..

[18] Lawrence M., Daujat S., Schneider R. (2016). Trends Genet..

[19] Chhabra R. (2015). ChemBioChem..

[20] Baulina N.M., Kulakova O.G., Favorova O.O. (2016). Acta Naturae..

[21] Boyko A., Smirnova N., Petrov S., Gusev E. (2016). Mult. Scler. Demyelinating Disord..

[22] Bramow S., Frischer J.M., Lassmann H., Koch-Henriksen N., Lucchinetti C.F., Sørensen P.S., Laursen H. (2010). Brain..

[23] Koch M., Kingwell E., Rieckmann P., Tremlett H. (2010). J. Neurol. Neurosurg. Psychiatry..

[24] Koch M., Kingwell E., Rieckmann P., Tremlett H. (2009). Neurology..

[25] Yadav S.K., Mindur J.E., Ito K., Dhib-Jalbut S. (2015). Curr. Opin. Neurol..

[26] Scalfari A., Neuhaus A., Degenhardt A., Rice G.P., Muraro P.A., Daumer M., Ebers G.C. (2010). Brain..

[27] Ascherio A., Munger K.L. (2007). Ann. Neurol..

[28] Afrasiabi A., Parnell G.P., Fewings N., Schibeci S.D., Basuki M.A., Chandramohan R., Zhou Y., Taylor B., Brown D.A., Swaminathan S. (2019). Genome Med..

[29] Niller H.H., Wolf H., Minarovits J. (2009). Semin. Cancer Biol..

[30] Hernán M.A., Olek M.J., Ascherio A. (2001). Am. J. Epidemiol..

[31] Wan E.S., Qiu W., Baccarelli A., Carey V.J., Bacherman H., Rennard S.I., Agusti A., Anderson W., Lomas D.A., DeMeo D.L. (2012). Human Molecular Genetics.

[32] Marabita F., Almgren M., Sjöholm L.K., Kular L., Liu Y., James T., Kiss N.B., Feinberg A.P., Olsson T., Kockum I. (2017). Sci. Rep..

[33] Marczylo E.L., Amoako A.A., Konje J.C., Gant T.W., Marczylo T.H. (2012). Epigenetics..

[34] Ito K., Lim S., Caramori G., Chung K.F., Barnes P.J., Adcock I.M. (2001). FASEB J..

[35] Munger K.L., Zhang S.M., O’Reilly E., Hernán M.A., Olek M.J., Willett W.C., Ascherio A. (2004). Neurology..

[36] Alonso A., Jick S.S., Olek M.J., Ascherio A., Jick H., Hernán M.A., Alonso Á., Jick S.S., Olek M.J., Ascherio A. (2005). Arch. Neurol..

[37] Runmarker B., Andersen O. (1995). Brain..

[38] Joshi S., Pantalena L.-C., Liu X.K., Gaffen S.L., Liu H., Rohowsky-Kochan C., Ichiyama K., Yoshimura A., Steinman L., Christakos S. (2011). Mol. Cell. Biol..

[39] Boyne D.J., Friedenreich C.M., McIntyre J.B., Stanczyk F.Z., Courneya K.S., King W.D. (2017). Cancer Causes Control..

[40] Piperigkou Z., Franchi M., Götte M., Karamanos N.K. (2017). Matrix Biol..

[41] Hollenbach J.A., Oksenberg J.R. (2015). J. Autoimmun..

[42] Patsopoulos N.A., Baranzini S.E., Santaniello A., Shoostari P., Cotsapas C., Wong G., Beecham A.H., James T., Replogle J., Vlachos I.S. (2019). Science.

[43] Ayuso T., Aznar P., Soriano L., Olaskoaga A., Roldán M., Otano M., Ajuria I., Soriano G., Lacruz F., Mendioroz M. (2017). PLoS One..

[44] Field J., Fox A., Jordan M.A., Baxter A.G., Spelman T., Gresle M., Butzkueven H., Kilpatrick T.J., Rubio J.P. (2017). Genes Immun..

[45] Pinto-Medel M.J., Oliver-Martos B., Urbaneja-Romero P., Hurtado-Guerrero I., Ortega-Pinazo J., Serrano-Castro P., Fernández Ó., Leyva L. (2017). Sci. Rep..

[46] Calabrese R., Valentini E., Ciccarone F., Guastafierro T., Bacalini M.G., Ricigliano V.A.G., Zampieri M., Annibali V., Mechelli R., Franceschi C. (2014). Biochim. Biophys. Acta..

[47] Kumagai C., Kalman B., Middleton F.A., Vyshkina T., Massa P.T. (2012). J. Neuroimmunol..

[48] Calabrese R., Zampieri M., Mechelli R., Annibali V., Guastafierro T., Ciccarone F., Coarelli G., Umeton R., Salvetti M., Caiafa P. (2012). Mult. Scler..

[49] Ramagopalan S.V., Dyment D.A., Morrison K.M., Herrera B.M., Deluca G.C., Lincoln M.R., Orton S.M., Handunnetthi L., Chao M.J., Sadovnick A.D. (2008). BMC Med. Genet..

[50] Nociti V., Santoro M., Quaranta D., Losavio F.A., De Fino C., Giordano R., Palomba N., Rossini P.M., Guerini F.R., Clerici M. (2018). PLoS One..

[51] Sokratous M., Dardiotis E., Bellou E., Tsouris Z., Michalopoulou A., Dardioti M., Siokas V., Rikos D., Tsatsakis A., Kovatsi L. (2018). J. Mol. Neurosci..

[52] Wagner M., Sobczyński M., Bilińska M., Pokryszko-Dragan A., Cyrul M., Kuśnierczyk P., Jasek M. (2017). J. Mol. Neurosci..

[53] Neven K.Y., Piola M., Angelici L., Cortini F., Fenoglio C., Galimberti D., Pesatori A.C., Scarpini E., Bollati V. (2016). BMC Genet..

[54] Handel A.E., De Luca G.C., Morahan J., Handunnetthi L., Sadovnick A.D., Ebers G.C., Ramagopalan S.V. (2010). J. Neuroimmunol..

[55] Dunaeva M., Derksen M., Pruijn G.J.M. (2018). Mol. Neurobiol..

[56] Olsen J.A., Kenna L.A., Tipon R.C., Spelios M.G., Stecker M.M., Akirav E.M. (2016). EBioMedicine..

[57] Liggett T., Melnikov A., Tilwalli S., Yi Q., Chen H., Replogle C., Feng X., Reder A., Stefoski D., Balabanov R. (2010). J. Neurol. Sci..

[58] Mastronardi F.G., Noor A., Wood D.D., Paton T., Moscarello M.A. (2007). J. Neurosci. Res..

[59] Slotkin R.K., Martienssen R. (2007). Nat. Rev. Genet..

[60] Bos S.D., Page C.M., Andreassen B.K., Elboudwarej E., Gustavsen M.W., Briggs F., Quach H., Leikfoss I.S., Bjølgerud A., Berge T. (2015). PLoS One..

[61] Souren N.Y., Gerdes L.A., Lutsik P., Gasparoni G., Beltrán E., Salhab A., Kümpfel T., Weichenhan D., Plass C., Hohlfeld R. (2019). Nat. Commun..

[62] Kular L., Needhamsen M., Adzemovic M.Z., Kramarova T., Gomez-Cabrero D., Ewing E., Piket E., Tegnér J., Beck S., Piehl F. (2019). Clin. Epigenetics..

[63] Huynh J.L., Garg P., Thin T.H., Yoo S., Dutta R., Trapp B.D., Haroutunian V., Zhu J., Donovan M.J., Sharp A.J. (2014). Nat. Neurosci..

[64] Maltby V.E., Lea R.A., Ribbons K.A., Sanders K.A., Kennedy D., Min M., Scott R.J., Lechner-Scott J. (2018). Mult. Scler. J. – Exp. Transl. Clin..

[65] Maltby V.E., Lea R.A., Sanders K.A., White N., Benton M.C., Scott R.J., Lechner-Scott J. (2017). Clin. Epigenetics..

[66] Rhead B., Brorson I.S., Berge T., Adams C., Quach H., Moen S.M., Berg-Hansen P., Celius E.G., Sangurdekar D.P., Bronson P.G. (2018). PLoS One..

[67] Ruhrmann S., Ewing E., Piket E., Kular L., Cetrulo Lorenzi J.C., Fernandes S.J., Morikawa H., Aeinehband S., Sayols-Baixeras S., Aslibekyan S. (2018). Mult. Scler..

[68] Graves M.C., Benton M., Lea R.A., Boyle M., Tajouri L., Macartney-Coxson D., Scott R.J., Lechner-Scott J. (2014). Mult. Scler..

[69] Baranzini S.E., Mudge J., van Velkinburgh J.C., Khankhanian P., Khrebtukova I., Miller N.A., Zhang L., Farmer A.D., Bell C.J., Kim R.W. (2010). Nature.

[70] Maltby V.E., Graves M.C., Lea R.A., Benton M.C., Sanders K.A., Tajouri L., Scott R.J., Lechner-Scott J. (2015). Clin. Epigenetics..

[71] Maltby V.E., Lea R.A., Graves M.C., Sanders K.A., Benton M.C., Tajouri L., Scott R.J., Lechner-Scott J. (2018). Sci. Rep..

[72] Kular L., Liu Y., Ruhrmann S., Zheleznyakova G., Marabita F., Gomez-Cabrero D., James T., Ewing E., Lindén M., Górnikiewicz B. (2018). Nat. Commun..

[73] Ewing E., Kular L., Fernandes S.J., Karathanasis N., Lagani V., Ruhrmann S., Tsamardinos I., Tegner J., Piehl F., Gomez-Cabrero D. (2019). EBioMedicine..

[74] Kulakova O.G., Kabilov M.R., Danilova L.V., Popova E.V., Baturina O.A. (2016). Acta Naturae..

[75] Chomyk A.M., Volsko C., Tripathi A., Deckard S.A., Trapp B.D., Fox R.J., Dutta R. (2017). Sci. Rep..

[76] https://www.uniprot.org/.

[77] https://www.ncbi.nlm.nih.gov/gene.

